# Acute upper limb arterial ischemia in patients diagnosed with COVID-19: case series

**DOI:** 10.1590/1677-5449.200234

**Published:** 2021-06-16

**Authors:** Felipe Damascena Rosa, Marcelo Calil Burihan, Elexandra Aparecida Simões, João Paulo de Souza Abdala, Orlando da Costa Barros, Felipe Nasser

**Affiliations:** 1 Hospital Santa Marcelina, São Paulo, SP, Brasil.; 2 Hospital Israelita Albert Einstein, São Paulo, SP, Brasil.

**Keywords:** COVID-19, anticoagulants, emboli and thrombosis, ischemia, prostaglandins

## Abstract

Infection by coronavirus 2, cause of the severe acute respiratory syndrome (SARS-CoV-2) in humans, was detected for the first time in Wuhan, China, in 2019, and spread globally over the course of 2020. Its different clinical manifestations are challenging, with a wide spectrum of presentations, ranging from asymptomatic infections to severe forms that can result in death. The objective of this study is to describe a series of four cases of acute arterial ischemia involving the upper limbs in patients diagnosed with COVID-19, which were managed clinically with anticoagulation, platelet antiaggregation, and prostanoids. Two patients were discharged from hospital with regression and delimitation of the ischemic zone, without needing surgical intervention, while two patients died from pulmonary complications. Adequate understanding of the pathophysiology of this disease could support better clinical management of its complications.

## INTRODUCTION

The emergence of a new disease caused by the severe acute respiratory syndrome coronavirus 2 (SARS-COV-2) in December 2019 in Wuhan, China, brought with it the challenge of achieving clinical and pathophysiologic characterization. The disease spread all over the world during the first half of 2020, was named the novel coronavirus disease (COVID-19) by the World Health Organization, which declared it a pandemic in March 2020.^1^

Initially presenting as a respiratory syndrome involving the lower respiratory tract, a large variety of signs, symptoms, and clinical presentations were soon observed, ranging from oligosymptomatic cases to cases that progress to respiratory insufficiency, coagulopathy, multiple organ failure, and death.^2^^,^^3^ Studies have identified a hypercoagulable state that can provoke complications in the micro and macro circulation.^4^^-^6

Below, a series will be described of four patients who exhibited acute arterial ischemia in the upper limbs and were diagnosed with COVID-19. This project was approved by the Research Ethics Committee CAAE 37208320.5.0000.0066, ruling number: 4.303.538.

## CASE REPORTS

### Case 1

**Figure 1:**
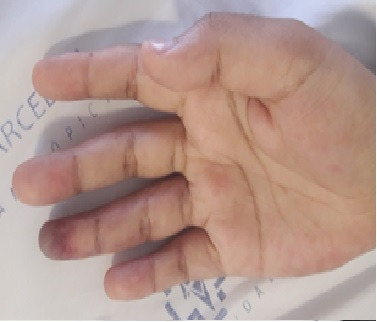
Cyanosis of the fourth finger of the right hand.

The patient was an 18-year-old, previously healthy, female who presented at the emergency room complaining of continuous pain of moderate and progressive intensity, associated with cyanosis of the fourth finger of the right hand, with onset 2 days previously. She denied fever, trauma, respiratory complaints, allergies, smoking, injected drug use, or contact with people diagnosed with COVID-19. She reported that she was taking a combined oral contraceptive regularly (levonorgestrel 0.15 mg and ethinylestradiol 0.03 mg). Physical examination was unremarkable, with the exception of upper limb palpation, during which pulses were present and symmetrical, but the distal phalanx of the fourth finger of the right hand had a lower temperature and had blanching cyanosis (Figure 1). The results of laboratory tests ordered at admission are shown in Table 1. Tests for rheumatoid factor and anti-DNA antibody were negative and a lupus anticoagulant test was positive. Vascular echography (VE) of the right upper limb showed patent arteries with normal caliber and triphasic flow and no abnormalities were provoked by maneuvers to identify thoracic outlet syndrome (TOS). A reverse transcriptase- polymerase chain reaction (RT-PCR) assay for SARS-CoV-2 in a nasopharyngeal sample was positive.

**Table 1:** Laboratory tests at admission.

	**Patient 1**	**Patient 2**	**Patient 3**	**Patient 4**
**Hemoglobin (g/dL)**	10.3	10.7	12.4	10.7
**Hematocrit (%)**	30.9	32.1	36.2	31
**Leukocytes/mL**	5,906	9,858	13,680	4,278
**Platelets/mL**	229,400	218,800	222,063	181,500
**INR**	1.08	1.37	1.2	1.32
**APTTr**	1.04	1.03	1.08	1.26
**PCR (mg/dL)**	11	15.9	35.3	115.2
**CPK**	35	65	260	106
**Urea (g/dL)**	28	50	39	90
**Creatinine (g/dL)**	0.52	1.24	0.6	1.41
**D-dimer (mcg/dL)**	1,750	-	1,825	881
**Glycemia (mg/dL)**	75	102	136	112
**Glycated HB (%/dL)**	4.8	-	10.8	-
**Sodium (mg/dL)**	131	142	141	138
**Potassium (mg/dL)**	4.1	3.6	3.9	5.1
**Calcium (mg/dL)**	9.4	8.4	8.9	8.5
**Magnesium (mg/dL)**	2.2	1.9	1.95	1.81

INR = international normalized ratio; APTTr = activated partial thromboplastin time relation; PCR = polymerase chain reaction; CPK = creatine phosphokinase; HB = hemoglobin.

**Figure 2:**
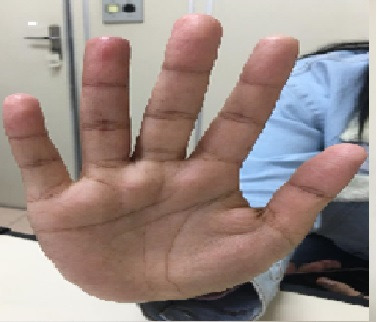
Fourth finger of the right hand after 14 days.

The patient underwent respiratory isolation and platelet antiaggregation was started with acetylsalicylic acid (ASA) at 100 mg/day, combined with full anticoagulation with enoxaparin (1 mg/kg every 12). Since the patient was still in pain, she was put on intravenous alprostadil (40 mcg every 12h) and prednisone at an anti-inflammatory dosage (0.5 mg/kg/day) for 7 days. She progressed with improvement in pain, regression of cyanosis, and resolution of the temperature gradient and was discharged from hospital on the 14th day after admission (Figure 2).

### Case 2

**Figure 3:**
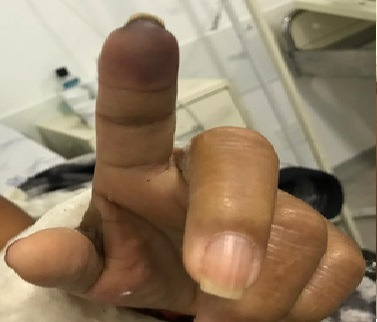
Non-blanching cyanosis of the second finger of the right hand.

The patient was a 57-year-old female with systemic arterial hypertension (SAH) and type 2 diabetes mellitus (DM) who presented at the emergency room complaining of intense and continuous pain in the second finger of the left hand that was unresponsive to standard analgesics, had onset 2 days previously and was associated with paresthesia, low temperature, and non-blanching cyanosis (Figure 3). She denied fever, trauma, coughing, use of injected drugs, and smoking. She stated that she was using glibenclamide, enalapril, ASA, cilostazol, and nifedipine regularly. General physical examination found nothing of note; palpation of the upper and lower extremities identified pulses present and symmetrical and a temperature drop at the level of the distal phalanx of the second finger of the left hand. The results of laboratory tests ordered at admission are shown in Table 1. VE of the left upper limb showed patent arteries with normal caliber and triphasic flow and no significant stenosis. No abnormalities were provoked by maneuvers to identify TOS. The echocardiogram showed normal ejection fraction, with no areas of ventricular asthenia or thrombi identifiable in the cardiac chambers.

**Figure 4:**
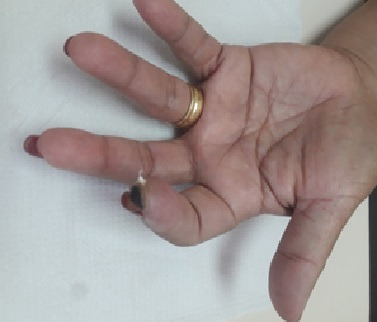
Dry necrosis, delimited, second finger of the right hand after 14 days.

The patient was put on full anticoagulation with enoxaparin (1 mg/kg every 12), combined with intravenous alprostadil (40 mcg every 12h) and prednisone at an anti-inflammatory dosage (0.5 mg/kg/day) for 7 days. The patient recovered well, but since the non-blanching cyanosis of the finger remained, she was maintained on oral anticoagulation with warfarin, targeting an international normalized ratio (INR) between 2 and 3, in order to monitor her progress in outpatients follow-up. She was discharged after 7 days in hospital on oral warfarin and with an INR of 2.15. At a 14-day outpatients visit, a small area of necrosis was found on the distal phalanx (Figure 4). Since a nasal swab had not been taken when she had been in hospital, a serological test for COVID-19 was ordered and the result was positive for IgG.

### Case 3

**Figure 5:**
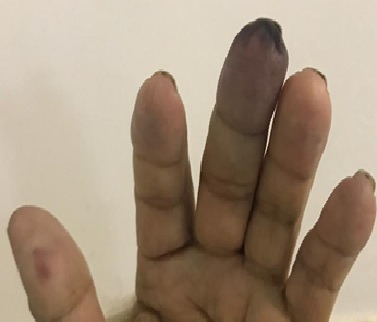
Non-blanching cyanosis of the third finger of the left hand.

**Figure 6:**
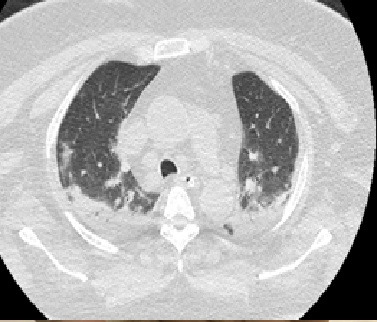
Axial computed tomography image of the chest, showing bilateral ground glass signs.

The patient was a 75-year-old female ex-smoker (40 pack-years) with grade III obesity, type 2 DM, SAH, and motor sequelae involving the left lower limb caused by a prior stroke. She presented at the emergency room complaining of pain in the left hand, with sudden onset 7 days previously. The pain was continuous and worsening progressively and had progressed to cyanosis of the fingers. She denied trauma, fever, coughing, dyspnea, or use of injected drugs. She was taking metformin, glibenclamide, insulin, atenolol, hydrochlorothiazide, phenytoin, and ASA. Physical examination found axillary, brachial, and ulnar pulses were present and normal, but the radial pulse was absent. The third finger had non-blanching cyanosis and a lower temperature and the remaining fingers of the left hand had blanching cyanosis (Figure 5). The results of laboratory tests ordered at admission are shown in Table 1. VE of the left upper limb showed patent arteries, with normal caliber and triphasic flow, with diffuse atherosclerosis and atherosclerotic occlusion of the proximal third of the radial artery. Maneuvers to identify TOS were negative. The echocardiogram showed a 61% ventricular ejection fraction, with no areas of ventricular asthenia or thrombi identifiable in the cardiac chambers. Computed tomography (CT) of the chest showed diffuse ground glass signs, compromising more than 50% of the pulmonary fields bilaterally (Figure 6). Her RT-PCR assay for SARS-CoV-2 was positive.

She was put on full anticoagulation with enoxaparin (1 mg/kg every 12h), ASA 100 mg/day, and intravenous alprostadil (40 mcg every 12h). Her severe respiratory condition deteriorated and she died after 27 days in hospital.

### Case 4

**Figure 7:**
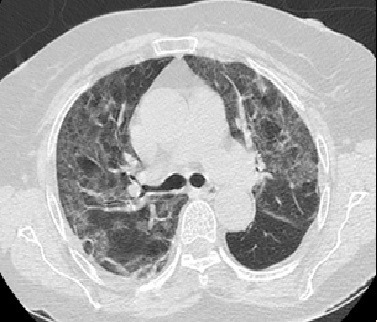
Axial computed tomography image of the chest, showing bilateral ground glass signs.

The patient was an 84-year-old female who presented at the emergency room with a history of dry coughing, general malaise, lack of appetite, and recurrent episodes of dyspnea, with onset 7 days previously. She described the following personal history: SAH and dyslipidemia, taking losartan, ASA, and simvastatin. Physical examination found diffuse rales throughout the entire right hemithorax and the lower third of the left hemithorax, respiratory rate was 24 inspirations per minute, oxygen saturation was 77% in room air and 92% with an oxygen mask (4 L/min). Palpation of upper and lower extremities detected pulses present and symmetrical, with no temperature gradient. The results of laboratory tests ordered at admission are shown in Table 1. An RT-PCR assay of a nasopharyngeal sample was positive for SARS-CoV-2. CT of the thorax showed diffuse ground-glass interstitial-alveolar opacities (Figure 7).

**Figure 8:**
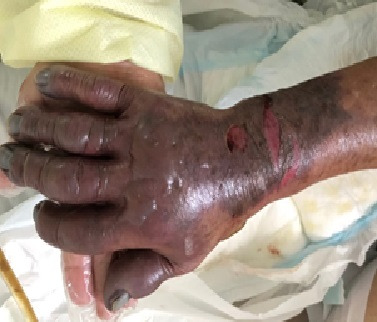
Right hand with non-blanching cyanosis, blistering, and distal pulses present.

The patient’s condition became critical and, on the 10th day after admission to the intensive care unit (ICU), she exhibited non-blanching cyanosis involving the right hand only, with pulses still present (Figure 8). She was put on a full anticoagulation protocol with unfractionated heparin, which contained the ischemia. However, the patient died from infectious and pulmonary complications after 42 days in hospital.

## DISCUSSION

The initial presentation of COVID-19 is usually with respiratory symptoms such as dry coughing, sore throat, and general nonspecific symptoms, such as asthenia, body pains, and fever.^2^^,^^3^ The most severe cases, requiring oral endotracheal intubation and developing septic shock and coagulopathy, generally occur during a later phase, between the 7th and 12th days of the disease, provoking a need for invasive support.^2^^,^^3^ Age over 60 years and presence of comorbidities such as obesity, DM, and SAH are recognized risk factors for unfavorable prognosis.^2^^,^^3^

The first three cases presented above did not develop typical clinical complaints and sought medical care because of microcirculation ischemia involving the upper limbs. Patients 1 and 2 had favorable clinical course and did not need interventions or prolonged hospital stays; anticoagulant treatment and antiplatelet drugs were started early, which could have contributed to preventing progression of the ischemic injuries.^7^

Tang et al.^4^ demonstrated that elevation of fibrin degeneration products, including D-dimer, and changes affecting the fibrinolytic system can be present from the initial phases of COVID-19, predisposing to pro-thrombotic states and worse prognosis.^5^^,^^6^ Anatomopathological studies have identified fibrin deposits in the alveoli and interstitial space in the lungs, with additional evidence of microcirculation thrombosis.^8^ Moreover, adoption of anticoagulation protocols has been associated with reduced mortality of patients with COVID-19.^9^^,^^10^ Presence of the lupus anticoagulant antibody has been observed in patients with COVID-19 and its possible relationship with the prothrombotic state is under investigation.^11^ It is still unclear whether the changes to hemostasis are directly caused by SARS-COV-2 or whether they are a consequence of an exacerbated inflammatory response, the “cytokine storm”.^12^ As such, use of heparin may contribute both because of its anticoagulant effect and because of its anti-inflammatory effect.^13^^-^17

Use of prostanoids in cases of critical ischemia without the conditions for revascularization has been described in the literature and there are previous reports of good results in terms of relief from pain and a small favorable effect on healing of wounds. There is also a case report of their use in the context of COVID-19.^18^^,^^19^ In the cases presented here, improvements in pain were observed after introduction of alprostadil, without adverse reactions.

An increase in cases of acute arterial ischemia has been reported during the pandemic. Bellosta et al.^20^ described 20 cases of acute arterial ischemia of lower limbs requiring surgical revascularization. Mortality was 40% and full anticoagulation during the postoperative period was related to better prognosis and a lower rate of reinterventions during the postoperative period.^20^ In this sample of patients, they did not observe major hemorrhagic events among the patients put on anticoagulation and outcomes were apparently good in relation to containment of ischemic phenomena.
