# Supplementary material for: Acute upper limb arterial ischemia in patients diagnosed with COVID-19: case series
Source: J Vasc Bras. 2021 Jun 16;20:e20200234. doi: 10.1590/1677-5449.200234 (PMC8218825; doi:10.1590/1677-5449.200234)
Supplement: Translation

# Translation: Isquemia arterial aguda de membros superiores em pacientes diagnosticados com COVID-19: série de casos

**Authors:** Felipe Damascena Rosa, Marcelo Calil Burihan, Elexandra Aparecida Simões, João Paulo de Souza Abdala, Orlando da Costa Barros, Felipe Nasser

**Affiliations:** 1 Hospital Santa Marcelina, São Paulo, SP, Brasil.; 2 Hospital Israelita Albert Einstein, São Paulo, SP, Brasil.

**Keywords:** COVID-19, anticoagulantes, embolia e trombose, isquemia, prostaglandinas

## Abstract

A infecção pelo coronavírus 2 causador da síndrome respiratória aguda grave (SARS-CoV-2) em humanos foi detectada pela primeira vez em Wuhan, na China, em 2019 e dispersada mundialmente ao longo de 2020. As diferentes manifestações clínicas, com amplo espectro de apresentação, desde infecções assintomáticas até formas graves que podem levar a óbito, são desafiadoras. Este trabalho objetiva descrever uma série de quatro casos de isquemia arterial aguda dos membros superiores em pacientes diagnosticados com COVID-19, os quais foram manejados clinicamente com anticoagulação, antiagregação plaquetária e uso de prostanoides. Dois pacientes receberam alta hospitalar com regressão e delimitação da área isquêmica, sem necessidade de intervenção cirúrgica, e dois pacientes faleceram em decorrência de complicações pulmonares. Uma adequada compreensão da fisiopatologia dessa doença pode favorecer um melhor manejo clínico de suas complicações.

## INTRODUÇÃO

O surgimento de uma nova doença causada coronavírus 2 causador da síndrome respiratória aguda grave (SARS-COV-2) em dezembro de 2019 em Wuhan, na China, que se espalhou ao redor do mundo no primeiro semestre de 2020, impôs novos desafios para a caracterização clínica e fisiopatológica dessa enfermidade. Denominada doença do novo coronavírus (COVID-19) pela Organização Mundial de Saúde, a doença foi declarada pandêmica em março de 2020^1^.

Inicialmente apresentada como síndrome respiratória de vias aéreas inferiores, logo se observou uma gama de sinais, sintomas e apresentações clínicas, que vão desde casos oligossintomáticos até àqueles que evoluem com insuficiência respiratória, coagulopatia, disfunção de múltiplos órgãos e morte^2^^,^^3^. Estudos têm apontado para um estado de hipercoagulabilidade, que poderia culminar em complicações da micro e da macrocirculação^4^^-^6.

A seguir, será relatada uma série de quatro pacientes que apresentaram isquemia arterial aguda dos membros superiores e diagnóstico de COVID-19. Este trabalho foi aprovado pelo Comitê de Ética em Pesquisa CAAE 37208320.5.0000.0066, número do parecer: 4.303.538.

## RELATO DE CASOS

### Caso 1

Paciente do sexo feminino, 18 anos de idade, previamente hígida, deu entrada no pronto-socorro com queixa de dor contínua, de moderada intensidade, progressiva, associada a cianose de quarto quirodáctilo direito, com início há 2 dias. Negava febre, trauma, queixas respiratórias, alergias, tabagismo, uso de drogas injetáveis ou contato com pessoas diagnosticadas com COVID-19. Referia fazer uso regular de anticoncepcional oral combinado (levonorgestrel 0,15 mg e etinilestradiol 0,03 mg). Ao exame físico, apresentava-se sem anormalidades, exceto na palpação das extremidades superiores, em que foram observados pulsos presentes, simétricos, redução da temperatura a nível de falange distal de quarto quirodáctilo direito e cianose não fixa (Figura 1). Os exames laboratoriais à admissão estão discriminados na Tabela 1. A pesquisa do fator reumatoide e do anticorpo anti-DNA foi negativa, e do anticoagulante lúpico foi positiva. A ecografia vascular (EV) do membro superior direito demonstrou artérias pérvias, de calibre preservado, com fluxo trifásico, sem alterações às manobras para identificação de síndrome do desfiladeiro torácico (SDT). O exame de reação da transcriptase reversa seguida pela reação em cadeia da polimerase (RT-PCR) para SARS-CoV-2 em amostra nasofaríngea foi positivo.

**Figura 1:** Cianose em quarto quirodáctilo direito.

**Tabela 1:** Exames laboratoriais à admissão.

	**Paciente 1**	**Paciente 2**	**Paciente 3**	**Paciente 4**
**Hemoglobina (g/dL)**	10,3	10,7	12,4	10,7
**Hematócrito (%)**	30,9	32,1	36,2	31
**Leucócitos/mL**	5.906	9.858	13.680	4.278
**Plaquetas/mL**	229.400	218.800	222.063	181.500
**RNI**	1,08	1,37	1,2	1,32
**rTTPA**	1,04	1,03	1,08	1,26
**PCR (mg/dL)**	11	15,9	35,3	115,2
**CPK**	35	65	260	106
**Ureia (g/dL)**	28	50	39	90
**Creatinina (g/dL)**	0,52	1,24	0,6	1,41
**D-dímero (mcg/dL)**	1.750	-	1.825	881
**Glicemia (mg/dL)**	75	102	136	112
**HB Glicada (%/dL)**	4,8	-	10,8	-
**Sódio (mg/dL)**	131	142	141	138
**Potássio (mg/dL)**	4,1	3,6	3,9	5,1
**Cálcio (mg/dL)**	9,4	8,4	8,9	8,5
**Magnésio (mg/dL)**	2,2	1,9	1,95	1,81

RNI = razão de normatização internacional; rTTPA = relação de tempo de tromboplastina parcial ativada; PCR = reação em cadeia da polimerase; CPK = creatinofosfoquinase; HB = hemoglobina.

Foi realizado isolamento respiratório e iniciada antiagregação plaquetária com ácido acetilsalicílico (AAS), 100 mg/dia, associado à anticoagulação plena com enoxaparina (1 mg/kg de 12 em 12 h). Devido à manutenção de queixas álgicas, associou-se alprostadil por via intravenosa (40 mcg de 12 em 12 h) e prednisona em dose anti-inflamatória (0,5 mg/kg/dia) por 7 dias. A paciente evoluiu com melhora da dor, regressão da cianose e do gradiente térmico, recebendo alta hospitalar no 14º dia de internação (Figura 2).

**Figura 2:** Quarto quirodáctilo direito após 14 dias.

### Caso 2

Paciente do sexo feminino, 57 anos de idade, portadora de hipertensão arterial sistêmica (HAS) e diabetes melito (DM) tipo 2, deu entrada no pronto-socorro com relato de dor intensa e contínua em segundo quirodáctilo esquerdo, não responsiva a analgésicos comuns, com início há 2 dias, associado à parestesia, redução de temperatura e cianose fixa (Figura 3). Negava febre, trauma, tosse, uso de drogas injetáveis ou tabagismo. Relatava fazer uso regular de glibenclamida, enalapril, AAS, cilostazol e nifedipina. Ao exame físico geral, não foram detectadas anormalidades; a palpação das extremidades superiores e inferiores identificou pulsos presentes, simétricos, com redução de temperatura a nível da falange distal de segundo quirodáctilo esquerdo. Os exames laboratoriais à admissão estão discriminados na Tabela 1. A EV do membro superior esquerdo demonstrou artérias pérvias, de calibre preservado, com fluxo trifásico, sem estenoses significativas e sem alterações às manobras para identificação de SDT. O ecocardiograma apresentou fração de ejeção preservada, sem áreas de adinamia ventricular ou trombos identificáveis em câmaras cardíacas.

**Figura 3:** Cianose fixa em segundo quirodáctilo direito.

Optou-se por anticoagulação plena com enoxaparina (1 mg/kg de 12 em 12 h), associado a alprostadil por via intravenosa (40 mcg de 12 em 12 h) e prednisona em dose anti-inflamatória (0,5 mg/kg/dia) por 7 dias. A paciente evoluiu bem; no entanto, devido à manutenção de cianose fixa de extremidade, optou-se por manter a anticoagulação oral com varfarina, objetivando uma razão de normatização internacional (RNI) entre 2 e 3, a fim de acompanhar a delimitação do processo ambulatorialmente. A paciente recebeu alta no 7º dia de internação, com varfarina oral e RNI de 2,15. No retorno ambulatorial em 14 dias, foi observada pequena necrose seca em falange distal (Figura 4). Como não foi realizada coleta de *swab* nasal durante a internação, solicitou-se sorologia para COVID-19, sendo detectado IgG positivo.

**Figura 4:** Necrose seca, delimitada, segundo quirodáctilo direito após 14 dias.

### Caso 3

Paciente do sexo feminino, 75 anos de idade, portadora de obesidade grau III, DM tipo 2, HAS, ex-tabagista (40 maços-ano), com sequela motora em membro inferior esquerdo devido a um acidente vascular encefálico isquêmico prévio. Compareceu ao pronto-socorro queixando-se de dor há 7 dias em mão esquerda, de início súbito, contínua, com piora progressiva e evolução para cianose de quirodáctilos. Negava trauma, febre, tosse, dispneia ou uso de drogas injetáveis. Relatava uso de metformina, glibenclamida, insulina, atenolol, hidroclorotiazida, fenitoína e AAS. Ao exame físico, apresentava-se com pulsos axilar, braquial e ulnar presentes e normais e pulso radial ausente, cianose fixa e redução da temperatura do terceiro quirodáctilo e cianose não fixa dos demais quirodáctilos à esquerda (Figura 5). Os exames laboratoriais à admissão estão discriminados na Tabela 1. A EV do membro superior esquerdo evidenciou artérias pérvias, de calibre preservado, com fluxo trifásico, com aterosclerose difusa e oclusão aterosclerótica da artéria radial em seu terço proximal, e as manobras para identificação de SDT foram negativas. O ecocardiograma apresentou fração de ejeção ventricular de 61%, sem áreas de adinamia ventricular ou trombos identificáveis em câmaras cardíacas. A tomografia computadorizada (TC) de tórax evidenciou imagens em vidro fosco difusamente, comprometendo mais que 50% dos campos pulmonares bilateralmente (Figura 6). O RT-PCR para SARS-CoV-2 foi positivo.

**Figura 5:** Cianose fixa de terceiro quirodáctilo esquerdo.

**Figura 6:** Tomografia computadorizada, corte axial de tórax, com imagem em vidro fosco bilateralmente.

Optou-se por iniciar anticoagulação plena com enoxaparina (1 mg/kg de 12 em 12 h), AAS 100 mg/dia, associado a alprostadil por via intravenosa (40 mcg de 12 em 12h). A paciente evoluiu com quadro respiratório grave e óbito após 27 dias de internação.

### Caso 4

Paciente do sexo feminino, 84 anos de idade, deu entrada no pronto-socorro com história de tosse seca, mal-estar geral, inapetência e episódios recorrentes de dispneia há 7 dias. A paciente relatou os seguintes antecedentes pessoais: HAS e dislipidemia, em uso de losartana, AAS e sinvastatina. Ao exame físico, apresentava crepitações difusas em todo o hemitórax direito e terço inferior de hemitórax esquerdo, frequência respiratória de 24 incursões respiratórias por minuto (irpm), saturação de oxigênio de 77% em ar ambiente e de 92% com máscara de oxigênio (4 L/min). Na palpação das extremidades superiores e inferiores, foram detectados pulsos presentes, simétricos, sem gradiente térmico. Os exames laboratoriais à admissão estão discriminados na Tabela 1. Foi realizado RT-PCR de amostra nasofaríngea, sendo positivo para SARS-CoV-2. A TC de tórax revelou opacidades interstício-alveolares em padrão vidro fosco difusamente (Figura 7).

**Figura 7:** Tomografia computadorizada, corte axial de tórax, com imagem em vidro fosco bilateralmente.

A paciente evoluiu com gravidade, e, no 10º dia de internação em unidade de terapia intensiva (UTI), apresentou cianose fixa somente em mão direita, com pulsos presentes (Figura 8). Foi iniciado protocolo de anticoagulação plena com heparina não fracionada, com contenção da isquemia. No entanto, a paciente foi a óbito por complicações infecciosas e pulmonares após 42 dias de internação.

**Figura 8:** Mão direita com cianose fixa, lesões bolhosas e pulsos distais presentes.

## DISCUSSÃO

A apresentação inicial da COVID-19 é usualmente com sintomas respiratórios, como tosse seca, dor de garganta, e sintomas gerais inespecíficos, como adinamia, dores no corpo e febre^2^^,^^3^. As formas mais graves, com necessidade de intubação orotraqueal, choque séptico e coagulopatia, ocorrem geralmente em uma fase mais tardia, entre o 7º e 12º dia de doença, levando à necessidade de suporte intensivo^2^^,^^3^. A idade maior que 60 anos e a presença de comorbidades, como obesidade, DM e HAS, são reconhecidos fatores de risco para prognóstico desfavorável^2^^,^^3^.

Os três primeiros casos apresentados não desenvolveram queixas clínicas típicas e procuraram assistência médica devido a quadro de isquemia de microcirculação dos membros superiores. As pacientes 1 e 2 tiveram evolução favorável, sem necessidade de intervenções e internação prolongada; o tratamento anticoagulante e antiagregante plaquetário foi iniciado precocemente, o que pode ter contribuído para impedir a progressão da lesão isquêmica^7^.

Tang et al.^4^ demonstraram que elevação de produtos da degradação da fibrina, entre eles o D-dímero, e alterações do sistema fibrinolítico podem estar presentes desde as fases iniciais da COVID-19, predispondo a estados pró-trombóticos e pior prognóstico^5^^,^^6^. Estudos anatomopatológicos identificaram deposição de fibrina nos alvéolos e espaços intersticiais dos pulmões, com evidências adicionais de trombose da microcirculação^8^. Por outro lado, a adoção de protocolos de anticoagulação foi associada à diminuição da mortalidade em pacientes com COVID-19^9^^,^^10^. A presença de anticorpo anticoagulante lúpico tem sido observada em pacientes com COVID-19, e investiga-se sua possível relação com estado pró-trombótico^11^. Ainda é incerto que as alterações na hemostasia sejam devido ao efeito direto do SARS-COV-2 ou se seria consequência da resposta inflamatória exacerbada, a chamada “tempestade de citoninas”^12^. Nesse sentido, o uso da heparina poderia contribuir tanto pelo seu efeito anticoagulante quanto pelo seu efeito anti-inflamatório^13^^-^17.

O uso de prostanoides em casos de isquemia crítica sem condições de revascularização é descrito na literatura, havendo relatos prévios de bons resultados na melhora da dor e pequeno efeito favorável na cicatrização de feridas. Além disso, há relato de caso de seu uso no contexto da COVID-19^18^^,^^19^. Nos casos apresentados, foi observado melhora das queixas álgicas após a introdução de alprostadil, sem reações adversas.

O aumento de casos de isquemia arterial aguda tem sido relatado durante esta pandemia. Bellosta et al.^20^ descreveram 20 casos de isquemia arterial aguda de membros inferiores, com necessidade de revascularização cirúrgica. A mortalidade foi de 40%, e a anticoagulação plena no pós-operatório foi relacionada a melhor prognóstico e menor necessidade de reintervenção no pós-operatório^20^. Nesta casuística, não se observaram eventos hemorrágicos maiores nos pacientes submetidos à anticoagulação, tendo aparentemente bom desfecho em relação à contenção dos fenômenos isquêmicos.
